# Awareness and Perceptions of Food Safety Risks and Risk Management in Poultry Production and Slaughter: A Qualitative Study of Direct-Market Poultry Producers in Maryland

**DOI:** 10.1371/journal.pone.0158412

**Published:** 2016-06-24

**Authors:** Patrick Baron, Shannon Frattaroli

**Affiliations:** 1 Department of Environmental Health Sciences, Johns Hopkins Bloomberg School of Public Health, Baltimore, Maryland, United States of America; 2 Department of Health Policy and Management, Johns Hopkins Bloomberg School of Public Health, Baltimore, Maryland, United States of America; Agricultural University of Athens, GREECE

## Abstract

The objective of this study was to document and understand the perceptions and opinions of small-scale poultry producers who market directly to consumers about microbial food safety risks in the poultry supply chain. Between January and November 2014, we conducted semi-structured, in-depth interviews with a convenience sample of 16 owner-operators of Maryland direct-market commercial poultry farms. Three overarching thematic categories emerged from these interviews that describe: 1) characteristics of Maryland direct-market poultry production and processing; 2) microbial food safety risk awareness and risk management in small-scale poultry production, slaughter and processing; and 3) motivations for prioritizing food safety in the statewide direct-market poultry supply chain. Key informants provided valuable insights on many topics relevant to evaluating microbial food safety in the Maryland direct-market poultry supply chain, including: direct-market poultry production and processing practices and models, perspectives on issues related to food safety risk management, perspectives on direct-market agriculture economics and marketing strategies, and ideas for how to enhance food safety at the direct-market level of the Maryland poultry supply chain. The findings have policy implications and provide insights into food safety in small-scale commercial poultry production, processing, distribution and retail. In addition, the findings will inform future food safety research on the small-scale US poultry supply chain.

## Introduction

### Direct-to-consumer agriculture in the US food system and Maryland

Direct-to-consumer, or direct-market, agriculture describes the alternative system of agricultural production that markets local food products through direct sales at on-farm retail stands, farmer’s markets, community-supported agriculture (CSA) programs, farm-to-table restaurants, local food hubs and other such outlets. Sales through direct-to-consumer agriculture channels have been rapidly increasing in recent years in the US food system. According to an analysis of data from the 2007 USDA Census of Agriculture, direct-to-consumer sales grew from $404 million to $1.2 billion between 1992 and 2007 [1]. This market growth was double that for total agricultural sales over the same period. Using different criteria (which include sales direct to local restaurants, institutions, and secondary retailers and distributors, as well as local consumers), sales of local agricultural goods in 2008 were estimated to be $4.8 billion [2]. From 2006–2013 the number of nationally registered farmers’ markets nearly doubled from 4,385 to 8,144 [1]. Though recent expansion of direct-market agriculture has been notable, direct-market sales account for a tiny portion (0.4% in 2007) of total agricultural retail in the US [2]. Food safety risk awareness and concern among US consumers has increased over the last two decades, potentially driven in part by increased media coverage of food safety outbreaks and other food safety issues [3].

Despite this increase, there is scant public health research on food safety in direct-market poultry supply chains. One qualitative study of California small-scale pastured poultry producers and marketers evaluated the perspectives and opinions of individuals within this commercial population on best management practices, value systems, and the benefits and challenges of small-scale pastured poultry production [4]. The body of research evaluating microbial food safety issues in the industrial-scale supply chain for poultry meat and other animal products cannot be extrapolated to the direct-market supply chains. There are many factors intrinsic to each supply chain that differentiate large and small poultry operations that also may be associated with important food safety outcomes, including microbial contamination of poultry products with pathogenic bacteria, such as antibiotic-resistant foodborne pathogens. The factors that differentiate small-scale direct-market poultry production and processing from the industrial scale have not been studied. By analyzing data obtained through semi-structured, open-ended interviews with Maryland commercial direct-market poultry suppliers, this pilot study will begin to address this gap in the literature. Specifically, this paper will: 1) Describe the landscape of Maryland direct-market poultry producers and their operations with specific attention to the factors that differentiate this direct-market supply chain from the industrial poultry supply chain, and which may be associated with food safety outcomes. 2) Document the opinions, awareness and perspectives of Maryland direct-market poultry producers about food safety risks, risk management strategies, motivations for minimizing food safety risks and other related issues in the statewide direct-market supply chain. To the best of our knowledge, this manuscript offers the first analysis of commercial poultry producers’ and processors’ perspectives on food safety and risk management in the US direct-market poultry supply chain in the literature.

## Methods

In order to document and describe the perspectives and opinions of owner-operators of commercial poultry production facilities in Maryland on these issues, we conducted a series of in-depth interviews with producers. We identified participants via publicly available commercial registries and documents, including the MDA Rabbit and Poultry On-Farm Processing Database, and more than 20 other commercial self-registries that promote and advertise direct-market agricultural producers in Maryland. As a secondary strategy, we used snowball sampling [5] to identify participants whose contact information was not available through the aforementioned sources. Participants were recruited via email or phone contact, and offered a $20.00 cash incentive to participate. The lead author conducted all of the interviews at places convenient for the interviewees (on-farm or in a coffee shop).

We developed an interview guide that included open-ended questions organized under the following three categories and eight sub-categories, described in Table 1. The questions were designed to elicit information and responses on the Maryland direct-market poultry supply chain, focusing on issues related to food safety risks in poultry production. The questions were developed using several different sources of information, including meetings with policymakers (MDA employees responsible for administering the Rabbit and Poultry On-Farm Processing Certification program), as well as informal conversations with three small poultry farmers in neighboring states who are professional contacts of the investigators. We also reviewed the existing food safety literature on the small-scale poultry supply chain [6, 7, 8], including the California study previously described [4] and the USDA-FSIS inspection criteria for poultry processing facilities [9].

**Table 1:** Topics and Issues Raised During In-Depth Interviews.

**Major Themes**		
Maryland Direct-Market Poultry Supply Chain	Food Safety in Small-Scale Poultry Production and Processing	Motivations for Maintaining Food Safety
**Sub-Themes**		
Defining the landscape of Maryland direct-market poultry farming	Perspectives on microbial food safety risks in direct-market poultry supply chain	Perspectives on personal and professional reputation in direct-market agriculture systems
Describing characteristics of small-scale poultry production related to microbial food safety issues	Comparing food safety issues between different models of poultry production and processing	Morals, ethics, and philosophy of small-scale poultry farming and direct-market agriculture
Economics of direct-market agriculture in Maryland	Practices and methods used to mitigate microbial food safety risks in small-scale poultry production and processing	

We transcribed the interviews verbatim with the interviewees’ permission, then coded and annotated the transcripts to highlight important topics and observed patterns in the data. We further analyzed these coded transcripts to assess the frequency and strength of particular themes, opinions and perspectives in accordance with standard qualitative data analysis techniques [10].

Informed oral consent was obtained and recorded prior to beginning the interview. Written consent was not considered to be necessary because of the low-risk nature of the research and because participants did not want to have to sign a piece of paper that they believed might compromise their anonymous participation in the study. Thus, oral consent was considered to be acceptable. The participant’s informed oral consent was obtained by reading a standard script approved by the Johns Hopkins Bloomberg School of Public Health Institutional Review Board, and was recorded by the researcher signing and dating at the bottom of the written script after the participant had agreed to proceed with the interview. Signed and dated oral consent forms were retained for each participant; these methods were approved by the same Institutional Review Board.

The Johns Hopkins Bloomberg School of Public Health Institutional Review Board reviewed these methods and approved this project.

## Results

We organized the results along the following four topics: attributes of the Maryland direct-market poultry supply chain; descriptions and perspectives of on-farm commercial poultry slaughter and processing; awareness and perceptions of food safety and risks related to microbial contamination in small-scale poultry production and slaughter; and motivations for maintaining food safety and quality control among direct-market poultry retailers.

### Enrollment and Recruitment

We invited 43 participants to be interviewed via email. Twenty-one people responded, and one declined to be interviewed. Of the remaining 21, four expressed interest but did not meet the inclusion criteria and we were unable to follow up with the last respondent. We completed interviews with sixteen respondents between January, 2014 and November, 2014. The interviews lasted between 30 and 120 minutes, and averaged 75 minutes. Table 2 describes the participants using key demographic variables.

**Table 2:** Demographics and Background Characteristics of Study Participants.

**Gender**	Female (6/16)
	Male (10/16)
**Racial Background**	White/Caucasian (16/16)
**Experience (years)**	Mean: 13.2; Range: 1–45
**Age (years)**	Mean: 44.0; Range: 26–63
**On-Farm Poultry Processing Status**	On-Farm Facility (13/16)
	Third Party Processor (3/16)
**Slaughter/Processing Certification Status**	MDA-Certified (8/16)
	USDA-Certified (3/16)
	Uncertified (5/16)
**Location (County)**	Frederick (5/16)
	Washington (3/16)
	Harford (3/16)
	Baltimore County (2/16)
	Garrett (1/16)
	Calvert (1/16)
	Wicomico (1/16)

### Direct-market poultry in Maryland

Participants described their poultry operations, their experiences in direct-market agriculture, as well as more general perceptions and opinions on the statewide direct-market poultry supply chain. Our questions were designed to highlight factors that participants considered to be unique to direct-market poultry production (and which distinguish small-scale from industrial-scale poultry operations), and the characteristics of the direct-market poultry supply chain that participants related to food safety.

#### Professional experience

Many participants were new to poultry farming, with most (11 of 16) reporting ten years or less experience in the business. At the time of the interviews, five participants were in their first 36 months of commercial agriculture production. Those with a prior work history (13 of 16) came from diverse professional backgrounds, including: school bus driver, fishing boat captain, senior officer at a federal regulatory agency, corporate sales, manager of a fast food restaurant, DJ and local radio personality, high school science teacher, veterinarian, and a military officer. The three remaining participants were self-described “lifelong farmers”, with no other career experience. A large minority (about one-quarter of participants) were experienced farmers, sometimes with decades of personal experience and a family history of farming in Maryland.

#### Economics of direct-market poultry production and agriculture

There was broad consensus among participants that the small-scale poultry production business was relatively inexpensive to enter, with low startup costs for animals and infrastructure, and that the practices for successfully raising small flocks of poultry livestock were easy to learn. Many farmers cited the 7–12 weeks to raise broiler poultry to a weight where they can be slaughtered and sold as relatively quick. Adding to the economic appeal, all agreed that there is a large demand for direct-market poultry among their consumer base. However, every participant described broiler poultry as a low-margin product for profit, even at price points significantly higher than those of supermarket poultry products. Despite the perception of poultry as a low-margin product, there was consensus among participants that the low overhead and startup costs, as well as the relatively simple skill sets required, makes direct-market poultry attractive for farmers, particularly those newer to farming, or growing a new business. Participants also spoke of another investment needed to make the direct-market poultry model work: a large commitment of time and energy.

Participants described diversified models for their agricultural production and sales. None of the participants sold poultry meat products exclusively; all raised other livestock (laying hens, ducks, geese, guinea fowl, lamb, beef and dairy cattle, swine, goats, alpaca, emu, horses, cats, and dogs) on the same property where broiler poultry were kept. Many participants also sold a variety of produce and animal products such as milk, cheese, eggs and processed meats such as bacon, sausage, and cured and dried meats.

#### Pastured poultry

Thirteen participants described their poultry production model as a “pasture-based” system in which poultry are kept in mobile housing and rotated through different paddocks of open pasture. The remaining three participants described keeping small flocks of poultry in permanent, stationary housing where they have daily access to the outdoors. Both of these direct-market models differ dramatically from the all-indoor, high-density and high-scale poultry production models typically employed in industrial-scale poultry production. Pasture-based systems involve rotating poultry over the same parcels of land over time in a structured land management system. Participants described this system as efficient and clean. They also described the rotating pasture system as one that kept animal manure from concentrating on their grazing lands, and kept the poultry livestock cleaner and healthier by constantly moving them away from their manure and onto fresh pasture. Many participants also explained that providing the livestock access to pasture where they can supplement their feed-based diet with more diverse forage helped to bring down feed costs and reduced disease risk among their flocks.

#### On-farm poultry processing

Most participants (13/16) maintain on-farm poultry slaughter and processing facilities that are integrated into their poultry production system. Participants described slaughtering and processing small flocks of poultry by hand as a relatively quick and easy process to learn. The on-farm slaughter was also described as producing a clean and safe product for sale, but the process takes time and practice to perform efficiently.

Most participants quoted the price of entry-level infrastructure, which generally included a stainless-steel table with a sink drain, a scalding tank, a motorized feather-plucker, a chilling tank, and a commercial freezer, at $1,500–3,000. Most, but not all, described this as an inexpensive investment to start an on-farm poultry processing facility capable of small-scale commercial operation. Many participants believed that the low costs and relatively simple set of skills and practices to learn explained in part why many farmers, particularly new farmers, chose to incorporate on-farm poultry processing into their direct-market agriculture production models. As with small-scale poultry production, participants noted that time and energy, not capital, were the main investment needed for processing which they described as labor-intensive, time-intensive, exhausting, and even depressing.

*“*…*if you look in the Bible*, *they rotated who was sacrificing and slaughtering animals for food*. *You know*, *it’s not good psychologically to do that every day…”*

All participants acknowledged that there is variability in the amount of capital that different Maryland on-farm poultry processors invest in their facility and equipment. This was commonly referred to as an “investment gap”, wherein farmers with less money to spend will purchase cheaper or used equipment, and may also construct and engineer functional alternatives to more expensive processing infrastructure out of repurposed or spare parts and materials. One participant who constructed a homemade feather-plucker out of a sawed-off plastic rain barrel and a repurposed lawn mower motor referred to his processing operation as “southern engineered”.

While all participants acknowledged the existence of an investment gap, there was some disagreement as to whether this gap affected microbial food safety and cross-contamination risks during processing. The most common opinion among participants was that the slaughter practices and the attention to quality control were much more important than the equipment for maintaining food safety during poultry processing. Most participants held the opinion that a conscientious processor could achieve acceptable food safety outcomes with a low-investment facility, but that maintaining food safety was simpler and easier with newer and more expensive equipment and infrastructure. Two participants expressed that the investment gap could compromise food safety during processing, with increased risk of contamination associated with low-investment facilities, but they also described workplace practices as much more important than infrastructure investment in determining food safety outcomes.

Three recurring and significant themes emerged when participants were asked about the reasons they chose to process their poultry on-farm, rather than seeking out a third-party processor. One common reason participants mentioned was a lack of available infrastructure; all participants noted that there are very few options in Maryland or the surrounding states for independent poultry producers to take their birds to be processed so they can be sold legally. All participants who used a third-party USDA-FSIS inspected processor took their poultry to the same facility. On-farm processing represents a realistic and cost-effective option for participants to legally process their poultry into a product for sale on the direct market.

The second reason cited for electing to process on farm was cost-control. Participants observed that poultry meat, already a low-margin product, became even more costly to produce when contracting with a third party to slaughter and process the livestock. The poultry farmers who employ on-farm slaughter processing described this model as way to control costs. Conversely, the three participants who elected to use third party processors did so because they felt the extra cost was worth the time and energy required to slaughter and process the birds themselves.

The third and most pronounced recurring theme participants cited was quality control and food safety. Most participants who processed their own birds described the on-farm processing model as an integral part of a different food safety risk management paradigm than that of the industrial-scale poultry producers. Specifically, participants described the industrial model as one that assumes a high amount of microbial pathogenic contamination of livestock and carcasses during production, slaughter, and processing. As a result, this system relied on chemical disinfection at critical control points during slaughter and processing to reduce the food safety risks of widespread microbial contamination, or “dirty in, clean out”.

In contrast, multiple participants used the phrase “clean in, clean out” to describe a different paradigm for small-scale poultry farmers. By integrating the best practices of small-scale poultry livestock husbandry with careful hand-work, rigorous quality control during slaughter and processing, meticulous biosecurity measures, and active animal health surveillance (quarantining or culling sick birds from a healthy flock and controlling outside contamination risks) the resulting process is a lower-risk, “cleaner” system throughout the production, processing and distribution continuum. Rather than assuming high levels of contamination that must be engineered out of the supply chain at critical control points (as in the industrial model), the “clean in, clean out” paradigm assumes low levels of contamination at all stages of production, and in the initial stages of slaughter and processing. This paradigm informs practices and food safety controls designed to maintain cleanliness (rather than to reduce widespread contamination) during production, processing and distribution in order to minimize risk and increase food safety.

Participants opined that the slow speed and manual labor involved with on-farm processing enables greater care and precision that avoids microbial cross-contamination of facilities, equipment and carcasses, and reduces the risk of contaminated consumer products. Participants also maintained the perception that slow, careful hand processing allows for more opportunities for enhanced quality control and visual inspection of the product and the processing line to prevent adulterated poultry meat from reaching the consumer. The shared perception among participants was that the relatively slow speed of the on-farm processing model allows direct-marketers to exercise a higher level of quality control through these dual mechanisms of accident reduction on the processing line, and early detection and remediation of cross-contamination issues. Participants maintained that this led to a higher level of food safety protection than would be possible at a more mechanized facility.

*“…there are just fewer eyes on each bird [at the industrial scale]*, *the number of actual inspections of each bird is so much lower*, *like 0*.*1% of what we do*…*Compared to what we do*…*you get three sets of eyes on each bird*, *and that’s so important [for food safety]*.*”*

Intertwined with the importance of slower speed on food safety/quality control was the significance of slaughtering and processing poultry by hand. Hand processing is both endemic and unique (within the poultry slaughter industry) to the current practices of small-scale on-farm poultry processing. Participants contrasted this system to the highly mechanized and largely automated industrial-scale poultry processing facilities. The consensus opinion was that hand processing afforded many more opportunities for avoiding accidents that may compromise food safety during processing (such as a gut rupture) and for quickly remediating accidents of this kind when they do occur.

“…*they [on-farm processors] are doing it by hand for the most part…each carcass is handled on its own*, *all the cutting is done by hand*. *So you don’t see the number of accidental cuts to the intestines and stuff like that*, *as you would in an automated system*. *Because for one you’re processing a fraction of the number of birds*, *and also you’re trying not to hurt yourself*…*So that hand work*, *compared to the automatic systems*, *reduces the amount of bacterial meat contamination you’re going to see on the whole*.*”*

On-farm poultry processors generally reported only processing their own livestock, although two processors described occasionally providing processing services for very small producers (usually neighbors) who were raising poultry for personal consumption, not retail. Participants described this exclusionary process as a “biosecurity” measure, designed to keep any contamination or disease-causing agents from other farms or off-farm environments from compromising their production or processing systems.

In general, participants’ observations on the issue of microbial food safety comprehensively identified the smaller scale of production and processing itself at the FSIS-exempt level of operation as a structural advantage for maintaining food safety and controlling risks of contamination within this supply chain. This comprehensive factor of scale was connected to the speed at which processing occurred, the time dedicated to inspection and quality control, the practices and methods (such as hand work) that are employed, and the infrastructure and equipment of on-farm poultry processing.

#### Food safety risk awareness and perceptions

We asked participants about their perceptions and opinions of microbial food safety risks in small-scale poultry production to document participants’ thoughts about the relevant factors associated with microbial food safety in the Maryland direct-market poultry supply chain. Many of the participants described their operation or a typical Maryland direct-market poultry operation in contrast to their perception of a typical industrial-scale poultry operation.

#### Risk perceptions associated with the direct-market poultry supply chain

Participants believed themselves and the statewide population of poultry direct-market producers to have high levels of awareness of microbial food safety risk issues associated with commercial poultry production and processing. They perceived livestock production and slaughter as a system with inherent food safety risks that must be mitigated in order to provide safe food to their customers. As one participant explained when asked about risks of pathogen contamination on poultry products:

*“…when you’re talking about an omnivore that eats grain*, *and what they can have in their intestines*, *even if it’s the highest quality*, *sure there’s concern*. *And that’s why we take our processing so seriously*, *to make sure that food is unadulterated*.*”*

Another participant put it more simply:

“*…chickens are still just a pretty dirty animal no matter what you do*.*”*

Interviewees conveyed a general perception that small-scale poultry slaughter and processing is a relatively simple system to control (compared to the industrial poultry processing systems) in terms of maintaining a clean facility and providing unadulterated poultry products to consumers, as described in the previous section.

Participants also expressed that the smaller scale of their operations reduces the impact of population exposure. If a contamination event does occur and adulterated products make it to the market, the exposure is easier to identify and control.

*“…the scale we’re doing things at*, *I mean…the risks to people generally are just so much smaller than for [an industrial-scale poultry retailer] if something does go wrong*.*”*

However, many interviewees acknowledged that a lack of comprehensive regulation at the FSIS-exempt level of Maryland poultry processing and retail created the potential for food safety problems. Most of their concerns were for “bad actors”, or individuals who did not place the same priority on quality control and cleanliness as they did. Interviewees expressed concern that inadequate regulations and enforcement regimes could enable these “bad actors” to introduce poultry products into the direct-market supply chain that could compromise food safety and the reputation of the producers within this market overall. However, most participants described this pool of “bad actors” as a very small portion of the people commercially engaged in this economy at the direct-market level in Maryland and they generally expressed confidence that the market would eliminate most operations producing inferior or unsafe products.

#### Antibiotic resistance and food safety in poultry production

Interviewees described a high level of concern when asked their opinion on the trends of antibiotic and antimicrobial resistance in foodborne pathogens, and the potential impact of these trends on the public’s health.

“…*the more things we eat that have antibiotics in them*, *the harder it is for us to fight off the resistant bugs that come down the food chain…things like MRSA and all sorts of horrible kinds of bacteria are out there now*, *and there are brand new ones all the time*.*”*

Participants associated these trends with practices used in the industrial poultry production system, particularly the use of prophylactic and sub-therapeutic antibiotics. They considered antibiotic resistance in foodborne pathogens to be a problem associated with the industrial poultry supply chain, not the direct-market chain. None of the participants used sub-therapeutic antibiotics in their poultry production system.

*“…I see (antibiotic resistance in the food supply chain) as a huge problem*. *And I think it comes exclusively out of big companies that are raising confinement poultry and feeding them antibiotics…You know*, *they’re basically eroding the tools of humanity to keep ourselves from getting sick*, *and they’re doing it at everyone’s expense*.”

Participants frequently mentioned feeling “insulated” from the problems of antibiotic resistance they associated with the industrial poultry supply chain. Use of a model that reduced or eliminated the need for antibiotic or antimicrobial inputs during poultry production; strict implementation of biosecurity measures during production and processing; use of non-pharmaceutical methods for animal disease control such as active disease surveillance and culling and quarantining of sick birds; and careful sourcing of day-old broiler chicks were all cited as strategies to avoid sub-therapeutic antibiotic use. Many participants also explained their decision to not use or to limit use of antibiotics in their livestock and poultry production as customer-driven. Direct-market consumers are only willing to pay higher prices for poultry that is antibiotic-free.

#### Motivations for maintaining food safety

Participants also shared personal motivations for maintaining food safety as equally important when describing food safety in their operations. They broadly expressed the opinion that direct-market poultry producers generally hold a higher personal standard for cleanliness and quality control than industrial suppliers. These motivations, inherent in most small-scale and direct-market poultry producers, generally inform practices that participants believe provide for a lower-risk supply chain compared to the industrial model.

#### Elevated personal responsibility for food safety and quality control

Participants discussed several related motivations for direct-market poultry producers to practice higher levels of personal responsibility for quality control and food safety in their products relative to the industrial-scale poultry suppliers.

*“…everyone that I know is actively involved in the process*. *And it’s usually the same person going down to sell the product at the farmer’s market…the responsibility and the accountability is really transparent…Which you don’t see in the industrial sector because it’s so compartmentalized*. *The grower is separate from the processor is separate from the distributor which is separate from the retailer*. *So you have people…who don’t really care about the other parts*…*of the supply chain*, *or feel responsible for them*.*”*

Participants frequently discussed personal contact with their customers, and cited wanting to keep their customers satisfied as a major motivation for taking personal responsibility for providing the safest food possible.

*“…small producers are more conscientious [than large producers] because they’re more in touch with their market*. *They’re more in touch with their consumers*. *It’s a face-to-face industry…It’s that customer contact you get with this scale that makes a difference*. *Otherwise it’s just a label in a supermarket*.*”*

Every participant also reported consuming their own products, and many participants mentioned this as a major motivating factor for keeping their products clean.

Direct-market farms are, generally speaking, small businesses that operate on tight financial margins, and depend on a reliable core base of customers to remain profitable. Participants in the direct-market supply chain described serving customers from tight-knit communities, wherein protecting one’s personal reputation for providing safe, high-quality food is critical to the survival of one’s business.

*“I don’t have some big lobby…backing me and spending money to protect me*. *If something goes wrong…this is my livelihood*, *this is my family’s farm…”*

Many participants emphasized the need to maintain a high level of trust and a good reputation for having safer, healthier food as a factor differentiating the direct-market poultry supply chain from its industrial-scale counterpart.

#### Transparency

Transparency, or freely offering information about the practices used in poultry production and slaughter and providing public access to the farm and processing environments, was a recurring theme among participants and was described as an integral aspect of the philosophy and practices that promote safer food products in this supply chain. This issue was seen as particularly important in Maryland because—from the perspective of many participants—this level of the poultry industry lacks comprehensive regulation.

*“…one of the things that the sustainable ag[riculture] community prides itself on is transparency*. *And that’s the one tomato we’re always throwing back at industrial ag[riculture]*, *is that they don’t let anyone behind those [expletive] doors*. *They wouldn’t want you to look behind those doors*. *They want you to look at the paperwork*.*”*

Transparency keeps small commercial operators “honest” and ensures they are using the practices desired by the most food-conscious consumers (those who would bother to ask in-depth questions of farmers and conduct a farm visit).

## Discussion

### Applications for public health research and policy

The overwhelming majority of research on the US food system has focused on the industrial-scale supply chain for poultry and other agricultural products. This study is, to the best of our knowledge, the first attempt to describe the models and practices in use in a statewide direct-market poultry supply chain. We believe this study also constitutes the first qualitative research to analyze the opinions and perspectives of the participants in this market on issues affecting food safety and public health. The data collected and analyzed for this project can provide important information and lay the groundwork for more in-depth research into the epidemiology of antibiotic-resistance, and other food safety and public health issues in the direct-market poultry supply chain for Maryland. This analysis may also be of use to public health research in other regional food systems and other agricultural paradigms, such as the direct-market supply chains for other livestock animals and animal products.

Policymakers in Maryland and many other states are in the beginning stages of developing effective food safety policies and regulatory strategies for these markets. For poultry, where the USDA has established an exemption to food safety inspection and regulation for small-scale, direct-market poultry producers, states are tasked with the difficult job of promoting small agricultural markets while protecting a growing number of consumers from potential food safety risks. The lack of public health research on these topics is a missed opportunity to inform this undertaking.

The analyses structurally differentiating Maryland’s large and small-scale poultry supply chains can be applied to inform future research into food safety risks in Maryland’s direct-market poultry system. These participants offer insights on the methods, models and characteristics of operations and their ideas of best practices for food safety in small-scale poultry production and processing. In particular, these interviews offer initial insights into the factors which may alter the risks of food supply chain contamination with antibiotic-resistant and susceptible foodborne pathogens in the direct-market supply chain as compared with the industrial poultry supply. This analysis can provide the groundwork for developing a more accurate and nuanced understanding of the structural factors affecting food safety outcomes among small scale producers. In the absence of reliable research defining microbial food safety risks in the direct-market poultry supply chain, the perspectives on these risks held by individuals with professional knowledge and experience can provide valuable insight to inform further investigations about this growing supply chain.

Findings regarding participants’ perspectives on personal motivations and values of direct-marketers related to food safety risks can also be applied to advance public health research. Further research on food safety issues involving direct-market agriculture systems will benefit from a deeper contextual understanding of the philosophical grounding and personal motivations of the commercial operators in this supply chain. Our analysis demonstrates that the philosophical perspectives of the direct-market supply chain often directly inform what practices, models and priorities are applied by the farmers themselves to food safety risk management in small-scale poultry production and processing. A contextual understanding of the priorities and choices of this routinely disregarded stakeholder population is important for public health research for these reasons; this study offers a potentially rich source of data on an understudied population of these stakeholders.

As all of the poultry producers included in this research run highly diversified operations and are also direct-marketers of many other agricultural products, we also view these opinions and perspectives are relevant beyond the Maryland poultry supply chain, and may be applicable to aspects of the regional direct-market supply chain for poultry and other agricultural products.

### Sample size and generalizability of qualitative findings

While a sample size of 16 may seem like a low number, a broader survey of the Maryland direct-market poultry supply chain has identified fewer than 70 active participants in the direct-market poultry supply chain who met the inclusion criteria for this study at this time this research was performed [11]. However, while this sample may be fairly representative of Maryland’s direct-market poultry supply chain, it is reasonable to assume that some issues related to both food safety risks and regulation may be considerably different in other regions of the US. However, it is possible that there may be a regional effect in the generalizability of these findings, wherein the issues and perceptions of those issues in comparable populations may be more similar in regions proximate to Maryland than in those more geographically distant. Further investigation into these topics should include states such as Delaware, Virginia, Pennsylvania, New Jersey and West Virginia to establish the validity of such a regional effect.

### Limitations and other areas for further research

The findings from this study have some limitations. There may be some response bias among our convenience sample of participants who volunteered to be interviewed. Given that an estimated 20–25% of the direct-market poultry producers were sampled as part of this study (as established by the broader survey of statewide poultry production we have administered), we view these findings as at least representative of a large portion of the overall population. However, further research should be conducted to establish the extent of the validity of these findings within Maryland. There is also the possibility of a social desirability bias among respondents, wherein participants may tend to overstate behaviors and responses that will be viewed favorably in a social setting, and may downplay or exclude responses that may be viewed negatively. With specific regard to participants’ opinions on issues of “bad actors” in the statewide supply chain, it is noteworthy that no participants considered their operations to be sub-standard, which could indicate a kind of sensitivity or social acceptance bias. However, the value of this data is not as an assessment tool for evaluating the food safety performance standards of individual farmers, but rather as a commentary on the statewide food safety policies, which participants considered to be rife with holes in enforcement and surveillance, creating opportunities for “bad actors” to slip through. Additionally, participants were generally commenting on the state of the entire statewide supply chain, opining that most direct-market poultry farmers would hold the same high standards for quality and food safety as themselves. However, most of the interviews were carried out on the participants’ farms, where interviewees would frequently show and demonstrate how they achieved many of the principles and ideals they mentioned during interviews. The concept of transparency as explained by participants helps to explain why sensitivity and/or social desirability bias in this study population is not likely to present any major issues for interpreting these data.

These data represent a spectrum of opinions and perspectives among a sample population of key informants, namely, commercially active direct-market Maryland poultry producers. These findings do not necessarily inform any food safety outcomes, and cannot be used on their own to evaluate food safety issues in the Maryland direct-market poultry supply chain. However, these data are of research value because they provide an informed starting point for further quantitative and microbiological assessments of statewide direct-market poultry supply chains to test the hypotheses put forward by this data set. Findings from this study can be compared to other data sets, including contemporary consumer attitudes and awareness towards food safety risk issues, to generate informed hypotheses to be tested in future quantitative public health research in this environment.
